# A case report of iatrogenic coronarocameral fistula after surgical aortic valve replacement: multimodality diagnosis and intravascular ultrasound-guided left main intervention

**DOI:** 10.3389/fcvm.2026.1796191

**Published:** 2026-06-18

**Authors:** Antonios Karanasos, Antonios Rigas Papapanagiotou, Periklis Davlouros, Grigorios Tsigkas

**Affiliations:** Department of Cardiology, University of Patras, Patras, Greece

**Keywords:** coronarocameral fistula, intravascular imaging, intravascular ultrasound, multimodality imaging, percutaneous coronary intervention

## Abstract

Coronary fistulas draining into the left ventricle (LV) are exceptionally rare, and left main (LM) donor vessels almost invariably connect to right-sided structures rather than the LV. We report the case of a 69-year-old gentleman with prior mechanical surgical aortic valve replacement (SAVR) and a left internal mammary artery–to–left anterior descending (LIMA–LAD) bypass graft who presented with new-onset heart failure and a newly reduced left ventricular ejection fraction (∼40%). Transthoracic echocardiography demonstrated a normally functioning mechanical aortic valve, moderate functional mitral regurgitation and a color jet near the aortic root initially interpreted as mild paravalvular regurgitation. Coronary angiography revealed severe, calcified LM ostial and bifurcation disease together with a discrete contrast communication from the LM to the LV. Three-dimensional transthoracic echocardiography reclassified the jet as a coronary fistula (LM-to-LV) anatomically distinct from the prosthetic sewing ring, while intravascular ultrasound (IVUS) confirmed heavy ostial/bifurcation calcification and a focal opening in proximal LM resembling a side-branch, corresponding to the fistulous segment. IVUS-guided LM–LCx percutaneous coronary intervention with two drug-eluting stents resulted in optimal stent expansion and apposition, with preserved competitive LAD flow via the LIMA graft, while the LM-to-LV communication remained angiographically modest. To our knowledge, this is the first reported iatrogenic LM–to–LV fistula after mechanical SAVR and highlights the pivotal role of multimodality imaging in distinguishing LM originating fistulas from paravalvular regurgitation or aorto–LV tunnels, and in guiding management by prioritizing safe LM revascularization while deferring targeted fistula closure pending assessment of shunt burden and clinical impact.

## 1 Introduction

Coronarocameral fistulas (CCFs) are uncommon communications between a coronary artery and a cardiac chamber or great vessel, with an estimated prevalence of approximately 0.002% in the general population and 0.1%–0.2% among patients undergoing coronary angiography. Drainage into the left ventricle (LV) is particularly rare within this spectrum, accounting for a small minority of reported fistulas (1, 2).

Acquired – including iatrogenic – CCFs have been described after cardiac surgery and other structural or electrophysiologic interventions. Coronary-to-LV fistulas have been reported following mitral valve surgery and septal myectomy for hypertrophic obstructive cardiomyopathy; in selected cases they have been successfully treated with transcatheter coil or device closure or have shown spontaneous closure on follow-up (3, 4). However, in these series the donor vessel is usually not the left main (LM) coronary artery; most arise from the LAD, left circumflex (LCx), or right coronary artery (1, 2, 5).

Within this context, LM-donor fistulas are themselves uncommon and typically drain to right-sided structures such as the right atrium, pulmonary artery, or superior vena cava rather than to the LV, emphasizing how unusual an LM-to-LV communication is (6, 7). Moreover, in patients with prosthetic aortic valves, color Doppler jets adjacent to the sewing ring can mimic or obscure a CCF, and careful multimodality correlation (selective coronary angiography, 3D transthoracic echocardiography, and cross-sectional imaging) is required to distinguish a true paravalvular leak or aorto-LV fistula from a coronary-origin tract (8, 9).

Against this background, we describe an iatrogenic LM-to-LV fistula identified a decade after mechanical surgical aortic valve replacement (SAVR), coexisting with severe, calcified LM disease. To our knowledge, no previous report has documented an iatrogenic fistula from LM draining directly into the LV in the post-SAVR setting, although there have been reports of LM originating fistulas draining to other structures, or post-surgical communications between aorta and LV or LV outflow tract (LVOT) (7, 10, 11). This case therefore highlights a rigorous multimodality diagnostic pathway and an intravascular-imaging–guided revascularization strategy tailored to the unique hazards of LM anatomy.

## 2 Case presentation

### 2.1 Case description

A 69-year-old man presented with progressive exertional dyspnea (New York Heart Association class III) and mild bilateral ankle edema. His medical history included mechanical SAVR and concomitant left internal mammary artery–to–left anterior descending (LIMA–LAD) bypass graft surgery, 10 years earlier. Additional comorbidities were hypertension and dyslipidemia. He had no prior history of myocardial infarction or overt heart failure, and previous echocardiographic assessments had reportedly shown preserved LV systolic function.

On admission, transthoracic echocardiography (TTE) demonstrated a well-seated mechanical aortic valve with normal leaflet motion and no evidence of prosthetic stenosis. A color Doppler jet was visualized near the aortic root and prosthetic sewing ring and was initially interpreted as mild paravalvular aortic regurgitation. LV systolic function was moderately reduced, with an estimated ejection fraction ∼40%, and there was moderate functional mitral regurgitation. In the absence of an obvious precipitating cause, further ischemic and structural evaluation was pursued given the *de novo* systolic dysfunction.

### 2.2 Diagnostic assessment

**Figure 1:**
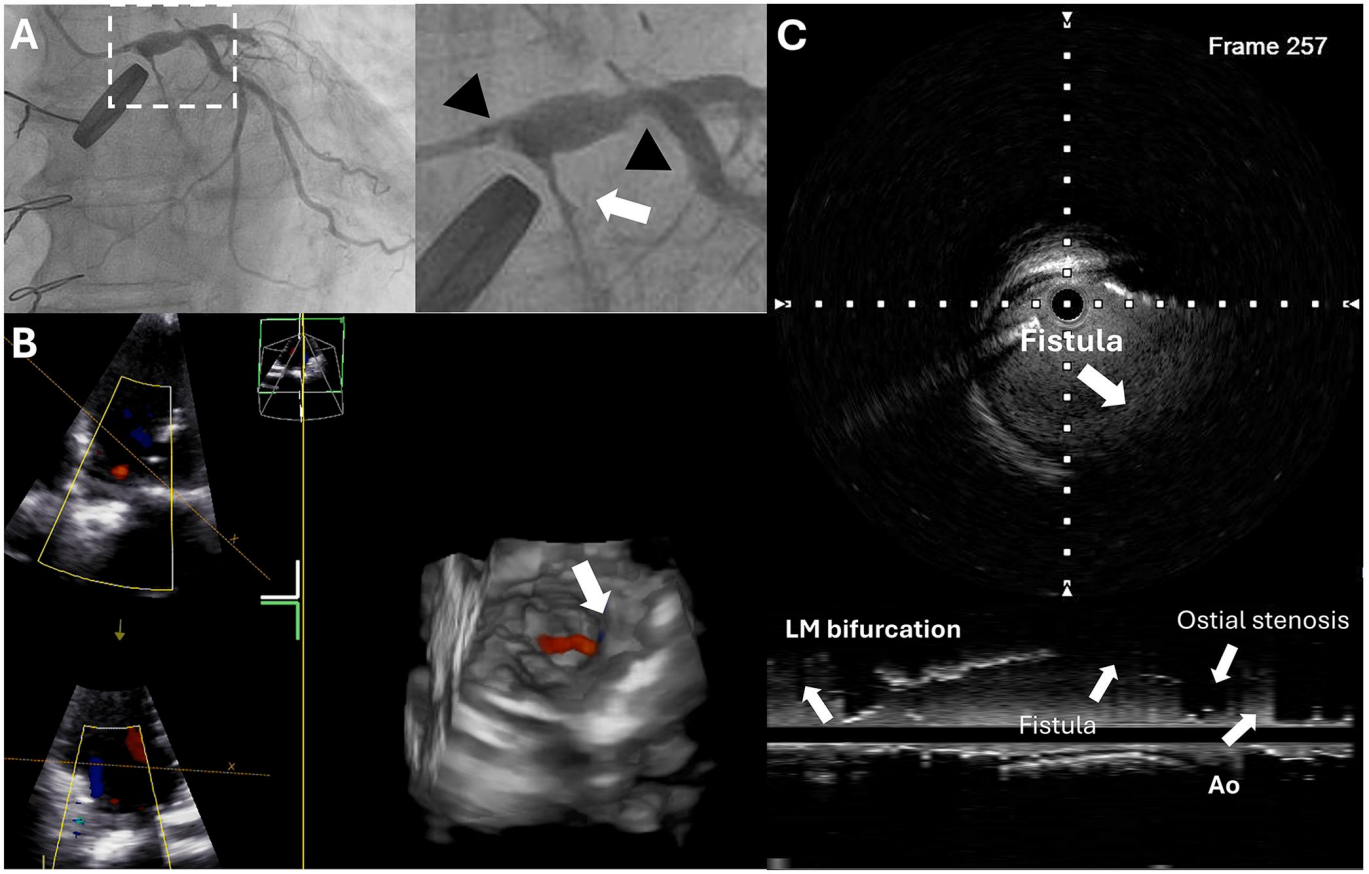
**(A)** angiographic image and magnification demonstrating the diseased left main (LM) and the presence of a coronarocameral fistula (arrow). Arrowheads indicate the sites of ostial and distal LM stenoses. **(B)** 3D transthoracic echocardiography (3D TTE) images derived from two non-orthogonal x-plane images showing flow (arrow) entering the left ventricle from a region outside the prosthetic valve or its annulus, anatomically consistent with the course of the LM artery. **(C)** Intravascular ultrasound (IVUS) image from the left main shaft, demonstrating an opening just distally to the stenosis of the LM ostium. Ao, aorta.

Invasive coronary angiography disclosed severe LM disease involving the ostium and bifurcation, with heavily calcified stenoses extending towards both the LAD and LCx origins (Figure 1A – Supplementary Videos 1, 2). The LIMA–LAD graft was patent and provided robust competitive flow to the LAD. The right coronary artery exhibited non–flow-limiting atherosclerotic disease. During selective LM injection, a discrete contrast jet was observed entering the LV cavity in close proximity to the aortic root, raising suspicion for a coronarocameral fistula originating from the LM.

Three-dimensional transthoracic echocardiography (3D TTE) from X-plane reconstruction was performed to further characterize this finding (Figure 1B – Supplementary Video 3). The available 3D/X-plane echocardiographic views and color Doppler directionality demonstrated that the jet visualized adjacent to the aortic root did not arise from the prosthetic sewing ring but instead originated from the region anatomically corresponding to the LM ostium and coursed directly into the LV cavity, consistent with a coronarocameral fistula (LM to LV). The mechanical aortic prosthesis functioned normally, with at most trivial true paravalvular regurgitation and no evidence of an aorto–LV tunnel or LV outflow tract defect. These echocardiographic findings corroborate the angiographic impression of an LM-derived fistulous tract, anatomically distinct from the prosthetic valve.

Based on these findings, the multidisciplinary heart team recommended intravascular ultrasound (IVUS)-guided percutaneous coronary intervention (PCI) of the LM as the initial therapeutic strategy, with planned multimodality imaging follow-up to reassess the hemodynamic impact of the fistula.

### 2.3 Intervention

**Figure 2:**
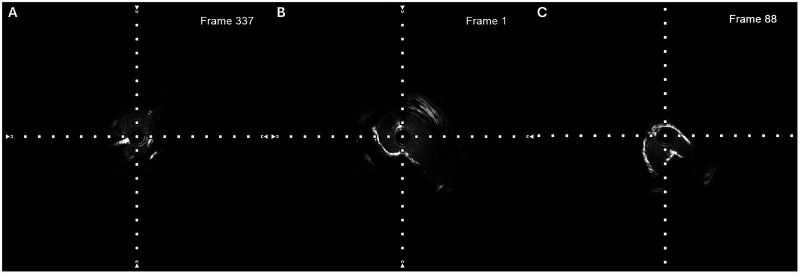
Intravascular ultrasound (IVUS) images from the left main (LM) coronary artery. **(A)** Ostial LM severe stenosis with a minimal lumen area of 5.5 mm². **(B)** Ostial LCx segment with marked plaque burden. **(C)** LM bifurcation showing heavily calcified stenosis with a protruding calcified nodule.

Intravascular imaging evaluation of the left main–to–left circumflex (LM–LCx) segment by IVUS confirmed severe, heavily calcified plaque at the ostial LM and at the LM–LCx bifurcation (Figure 2). At the left main shaft, distally to the ostial stenosis, there was an opening resembling a side-branch, appearing concordant with the angiographically suspected fistulous tract (Figure 1C – Supplementary Video 3). No intraluminal thrombus or dissection was identified, supporting a chronic, structurally integrated LM-to-LV communication rather than an acute catheter-induced perforation.

**Figure 3:**
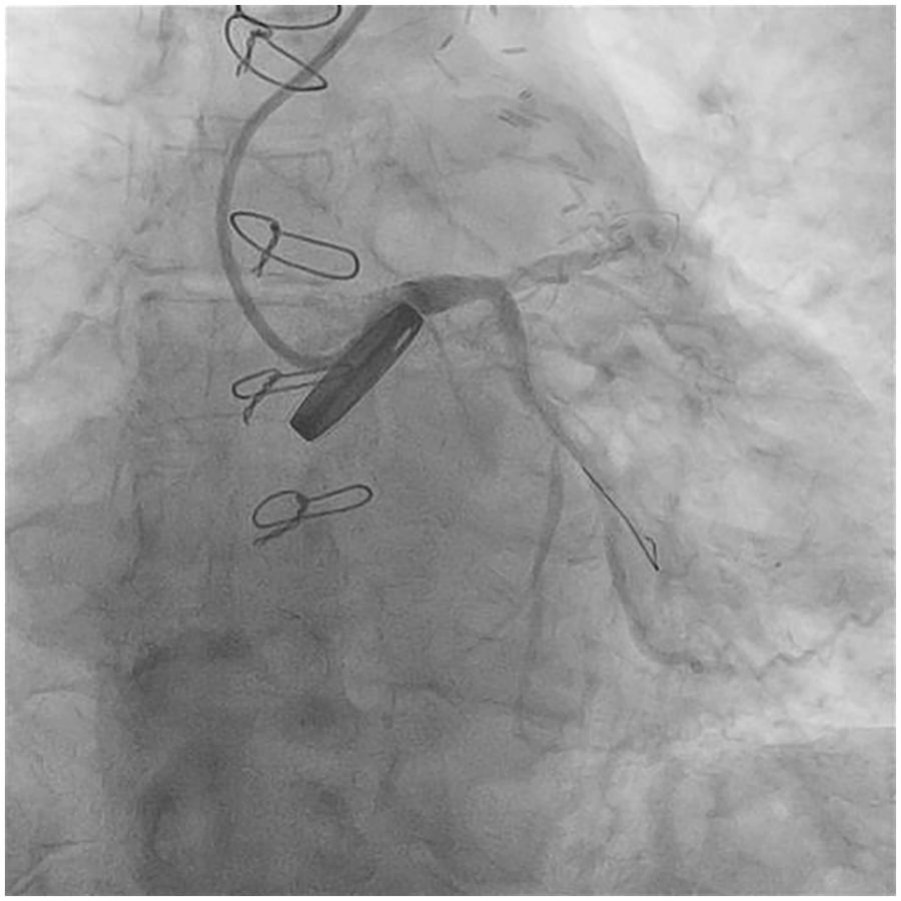
Final post-procedural angiographic result.

Guided by IVUS measurements and reference diameters, PCI from LM to LCx was performed with implantation of two drug-eluting stents to scaffold the ostial and bifurcation disease, followed by post-deployment high-pressure optimization. Final angiography demonstrated an excellent result in the LM–LCx segment, with TIMI 3 flow and preserved competitive perfusion of the LAD via the patent LIMA graft. The angiographic appearance of the LM-to-LV fistulous tract remained unchanged (Figure 3 – Supplementary Video 4).

### 2.4 Differential diagnosis and diagnostic reasoning

In a patient with a prior mechanical surgical aortic valve replacement and a newly observed color Doppler jet adjacent to the aortic root, the initial differential diagnosis included paravalvular aortic regurgitation, an aorto–LV or LVOT tunnel, and less commonly a coronarocameral fistula. Because prosthetic valves can generate complex flow artifacts and eccentric jets, careful anatomic localization of the jet origin and its downstream trajectory was essential to avoid mislabeling a coronary-origin fistula as prosthetic valve dysfunction.

Selective LM angiography demonstrated a discrete contrast-filled tract arising from the LM coronary artery and entering the LV cavity, strongly suggesting a coronarocameral communication rather than a paravalvular leak or aorto–LV tunnel. Three-dimensional transthoracic echocardiography corroborated the diagnosis through multiplanar reconstruction and color Doppler directionality which showed that the jet visualized near the aortic root originated from the region of the LM ostium, had intramyocardial course, and entered the LV cavity away from the prosthetic sewing ring. The mechanical valve leaflets moved normally, and only trivial true paravalvular regurgitation was present, effectively excluding clinically relevant paravalvular leak or a discrete aorto–LV defect.

Finally, IVUS added intraluminal context by demonstrating that the fistula originated distal to the LM origin concordant with the angiographic images, without evidence of acute dissection, perforation, or pseudoaneurysm formation. Taken together, these findings supported the diagnosis of a chronic LM-to-LV coronarocameral fistula, anatomically distinct from the mechanical aortic prosthesis and unrelated to an acute procedural complication.

### 2.5 Management rationale

Overall, the patient had presented with symptomatic *de novo* LV systolic dysfunction in the background of severe, calcific LM disease and concomitant LM-to-LV fistula of modest significance by angiography. As such, available options included: 1) simultaneous management of both conditions; 2) management of obstructive CAD first and staged reevaluation for potential closure of the fistula; and 3) closure of the fistula and subsequent reevaluation of the obstructive CAD. The Heart Team had taken several factors into consideration: 1) The patient had a high surgical risk due to advanced age and prior sternotomy with mechanical SAVR; 2) LM stenosis was significant by IVUS with MLA < 6.0 mm^2^ and >75% area stenosis; 3) The angiographic magnitude of the fistula was modest and of uncertain hemodynamic significance; 4) LM intervention could potentially hamper future attempts for fistula closure, but could as well reduce its significance by scaffolding of adjacent diseased segments potentially causing plaque shift, thus influencing the fistulous course or entry geometry, and stabilizing local flow dynamics by enhancing downstream flow; and 5) Percutaneous fistula closure by covered stent implantation or coil embolization could further introduce risks of residual communication from the aorta to the fistula behind the covered stent, and of device migration or inadvertent coronary flow compromise that can accompany transcatheter embolization from a LM donor to the LV. Therefore, IVUS-guided LM revascularization was prioritized to secure epicardial flow and reduce the risk of ongoing ischemia or supply–demand mismatch, with fistula closure reserved for follow-up if persistent symptoms, lack of LV functional recovery, or subsequent multimodality imaging suggested a hemodynamically significant shunt.

Invasive physiological assessment of the LM lesion was considered but not pursued, due to the presence of significant LM stenosis by IVUS (MLA < 6.0 mm^2^ and >75% area stenosis) and also challenges in interpretation due to aorto-ostial lesion location, concomitant LM-to-LV fistula, and competitive LAD flow from the patent LIMA graft.

### 2.6 Outcome and follow-up

Post left main PCI, the final angiogram showed an excellent stent result with TIMI 3 flow, no angiographic or access-site complications, and no change in opacification of the LM-to-LV fistulous tract. The patient remained clinically stable and asymptomatic during a three-day index hospitalization, during which guideline-directed medical therapy for coronary artery disease and heart failure was optimized.

He was discharged in good condition with plans for structured surveillance: repeat TTE at 3 months to reassess LV function, valvular performance, and any indirect impact of the fistula, and repeat coronary angiography at 6 months to evaluate stent patency and reassess the LM–LV communication. In the absence of symptomatic or echocardiographic improvement, further functional assessment of the fistula, including cardiac magnetic resonance (CMR)-based quantification, will be considered.

## 3 Discussion

Coronarocameral fistulas are rare coronary anomalies, with population-based and catheterization-based estimates placing their prevalence at approximately 0.002% and 0.1%–0.2%, respectively. Most fistulas originate from the right coronary or the left anterior descending artery and drain into right-sided chambers or the pulmonary artery, whereas LV-draining fistulas represent one of the least frequent configurations (1, 2). Within this spectrum, LM donor fistulas are distinctly uncommon and, when present, typically drain to right-sided structures such as the pulmonary artery, right ventricle, right atrium, or superior vena cava (7, 11). These epidemiologic patterns underscore how unusual a LM-to-LV communication is.

Congenital LM to LV or left-coronary-to-LV fistulas have been described in isolated case reports and small series, frequently delineated by a combination of echocardiography, CT angiography, and invasive angiography, and in some instances associated with ventricular dilation, ischemia, or heart failure symptoms (2, 5). However, these reports provide limited practical guidance when the donor is the LM, particularly in the setting of concomitant epicardial coronary artery disease.

By contrast, acquired and iatrogenic coronary-to-LV fistulas have been reported after valve surgery (especially mitral valve replacement) and septal myectomy for hypertrophic obstructive cardiomyopathy (3–5). Transcatheter coil or plug closure has been successfully employed in symptomatic or hemodynamically significant fistulas after mitral surgery, while spontaneous closure has been documented after septal myectomy, underscoring a heterogeneous natural history and supporting individualized decision-making in small or modest shunts (3, 4).

After aortic valve surgery, reported fistulous complications more often involve aorto-LV or LVOT-to-atrium tracts related to paravalvular pseudoaneurysm or annular disruption rather than true LM-origin cameral connections (10). LM-donor fistulas in the literature typically drain into right-sided structures (pulmonary artery, right ventricle, right atrium, or superior vena cava) and are occasionally associated with coexisting LM stenosis or multivessel coronary disease (7, 11). Taken together, the available data support the rarity of an iatrogenic LM-to-LV fistula in the post-SAVR setting, as illustrated in our patient. The delayed detection a decade after SAVR, absence of prior imaging evidence, and the anatomical course adjacent to the prosthetic sewing ring collectively favor an acquired (iatrogenic) rather than congenital origin, although the exact timing of fistula formation cannot be definitively established.

This case also emphasizes a multimodality, stepwise diagnostic strategy tailored to postsurgical anatomy. Selective LM angiography was pivotal in demonstrating discrete contrast opacification of the LV originating from the LM, a pattern not compatible with peri-annular extravasation or a simple aorto-LV paravalvular jet (2, 12). Three-dimensional transthoracic echocardiography then re-characterized the color jet initially suspected as paravalvular as a coronary origin tract (LM→LV) by localizing the origin distally to the LM ostium, confirming its intracavitary trajectory, and verifying normal mechanical SAVR function, thereby avoiding misclassification as paravalvular regurgitation. The importance of meticulous echocardiography–angiography correlation and cross-sectional imaging in CCF is well recognized and is crucial to prevent unnecessary valve reinterventions (2).

Intravascular ultrasound added intraluminal anatomic context, demonstrating a focal opening in the LM, distal to the LM ostial stenosis, co-localizing with the angiographically suspected tract and excluding an acute injury. Beyond its role in characterizing the fistulous segment, IVUS is central to the contemporary management of complex LM disease, guiding stent sizing, lesion preparation, and optimization (13).

Assessment of the hemodynamic relevance of a coronary fistula is also essential when deciding between conservative management and closure. Cardiac catheterization with oximetric Qp/Qs calculation remains a classical reference method for quantifying left-to-right shunts; however, its diagnostic value is limited in a LM-to-LV communication, where the shunt is left-to-left and no meaningful oximetric step-up is expected. In this setting, cardiovascular magnetic resonance with phase-contrast flow mapping may provide a more suitable non-invasive method for integrated follow-up assessment, allowing reassessment of LV volumes, systolic function, regional wall motion, and indirect quantification of fistula-related flow by comparing LV stroke volume with ascending aortic flow (14). CMR was not performed during the index hospitalization, which represents a limitation of the present report, but it may be considered during follow-up if symptoms persist or LV functional recovery remains incomplete.

Therapeutically, decision-making in CCF must balance three domains: (i) shunt significance (symptoms, LV volume loading or ischemia/steal), (ii) anatomic feasibility and risk to donor-vessel flow, and (iii) coexisting epicardial coronary disease. Contemporary reviews and expert consensus documents generally recommend closure of moderate-to-large or hemodynamically significant fistulas—particularly those associated with heart failure, myocardial ischemia, ventricular dilation/dysfunction, or prior endarteritis—using surgery or transcatheter techniques depending on anatomy (1, 2, 9, 12). Conversely, small, hemodynamically trivial fistulas can be managed conservatively with periodic imaging and clinical follow-up, especially when the procedural risks of closure are non-negligible (1, 2, 5, 12).

For LM donor fistulas specifically, the stakes of transcatheter closure are potentially higher: coils, plugs, or covered stents may compromise LM or branch flow, increase the risk of thrombosis, or prove unstable in the setting of high-flow, short-segment fistulas (7, 12). In such anatomies, a staged strategy is reasonable. First, epicardial flow is secured—here by IVUS-guided PCI of the severely calcified LM ostial/bifurcation disease, in the presence of a protected LAD territory from a patent LIMA graft—thereby reducing ischemic risk and stabilizing LM hemodynamics. Subsequently, the fistula's anatomy and physiology can be reassessed with echocardiography, selective angiography, and, where available, CMR to determine whether definitive closure is warranted. The observation that some acquired coronary-to-LV fistulas undergo spontaneous closure further supports an individualized, surveillance-based approach when the shunt is modest and symptoms are controlled (4, 5).

In our patient, the dominant clinical issue was severe, calcific LM disease in the setting of *de novo* LV dysfunction, with the LM-to-LV fistula appearing angiographically modest and not clearly responsible for the heart failure presentation. IVUS-guided LM–LCx stenting therefore addressed the principal ischemic substrate while preserving LM patency and avoiding the added risk of device deployment within or across the fistulous origin. The unchanged angiographic appearance of the LM-to-LV tract post PCI, combined with clinical stability, justified a strategy of structured follow-up (echocardiography at 3 months and invasive and/or CMR reassessment at 6 months) before reconsidering targeted closure. This approach is consistent with current principles for CCF management and reflects the unique procedural risk profile of LM-donor fistulas (1, 2, 9, 12).

In summary, this case extends the phenotype of acquired CCFs by documenting, to our knowledge, the first iatrogenic LM-to-LV fistula after mechanical SAVR, and it proposes a pragmatic, imaging-anchored framework: (i) confirm coronary-origin flow with selective angiography; (ii) use advanced echocardiography (and transesophageal echocardiography when appropriate) to distinguish coronary-origin jets from paravalvular regurgitation; (iii) leverage intravascular imaging to characterize LM anatomy and guide revascularization; and (iv) individualize fistula closure according to shunt burden, symptoms, and LM-specific procedural risk. Algorithmic approaches to CCF diagnosis and management increasingly emphasize exactly this integration of multimodality imaging and tailored intervention, and our case illustrates how these principles can be applied in a technically demanding left main scenario.

## Data Availability

The original contributions presented in the study are included in the article/Supplementary Material, further inquiries can be directed to the corresponding author.
